# Evaluation of the performance of national health systems in 2004-2011: An analysis of 173 countries

**DOI:** 10.1371/journal.pone.0173346

**Published:** 2017-03-10

**Authors:** Daxin Sun, Haksoon Ahn, Tomas Lievens, Wu Zeng

**Affiliations:** 1 School of Transportation and Civil Engineering, Fujian Agriculture and Forestry University, Fuzhou, Fujian, China; 2 School of Social Work, University of Maryland, Baltimore, MD, United States of America; 3 Oxford Policy Management, Oxford, United Kingdom; 4 Schneider Institutes for Health Policy, Heller School, Brandeis University, Waltham, MA, United States of America; Universidad Veracruzana, MEXICO

## Abstract

In an effort to improve health service delivery and achieve better health outcomes, the World Health Organization (WHO) has called for improved efficiency of health care systems to better use the available funding. This study aims to examine the efficiency of national health systems using longitudinal country-level data. Data on health spending per capita, infant mortality rate (IMR), under 5 mortality rate (U5MR), and life expectancy (LE) were collected from or imputed for 173 countries from 2004 through 2011. Data envelopment analyses were used to evaluate the efficiency and regression models were constructed to examine the determinants of efficiency. The average efficiency of the national health system, when examined yearly, was 78.9%, indicating a potential saving of 21.1% of health spending per capita to achieve the same level of health status for children and the entire population, if all countries performed as well as their peers. Additionally, the efficiency of the national health system varied widely among countries. On average, Africa had the lowest efficiency of 67%, while West Pacific countries had the highest efficiency of 86%. National economic status, HIV/AIDS prevalence, health financing mechanisms and governance were found to be statistically associated with the efficiency of national health systems. Taking health financing as an example, a 1% point increase of social security expenses as a percentage of total health expenditure correlated to a 1.9% increase in national health system efficiency. The study underscores the need to enhance efficiency of national health systems to meet population health needs, and highlights the importance of health financing and governance in improving the efficiency of health systems, to ultimately improve health outcomes.

## Introduction

It is widely recognized that countries face resource constraints for providing health services to their populations. The constraints are more prominent in low- and mid-income countries (LMICs), where health expenditure per capita is significantly lower than that in high-income countries, but the burden of diseases is much higher. According to statistics from national health accounts (NHA) in 2011[1], there were 32 countries where health spending per capita was lower than $60/year, the minimal cost to provide the basic package of health services recommended by World Health Organization (WHO) [2]. Many of those countries encountered enormous challenges to meet their population’s basic health care needs. As a result, many of them could not achieve the Millennium Development Goals (MDGs) on maternal and child health.

In the post MDG era, governments and international development partners are committed to sustainable development goals (SDGs) and Universal Health Coverage (UHC). To achieve the health-related goals, it requires not only generating more financial resources for health—“more money for health”, but also using available resources more effectively and efficiently—“more health for the money”. In countries where resources are limited, improving efficiency of health systems becomes more important to address health service delivery challenges and move towards UHC, as reflected in fiscal space analyses in several LMICs [3, 4]. Similarly, donors and international organizations are paying an increasing attention to efficiency issues in the health system. In 2010 World Health Report, WHO identified 10 leading sources of inefficiency in health systems, and estimated that “20–40% of total spending was consumed in ways that do little to improve people’s health”, calling for improvements in efficiency of national health care systems and better use of available funding[5, 6]. In 2012, the United States Agency of International Development (USAID) established the Office of Health Systems to help USAID funding recipient countries better use resources for health, and maximize the benefit of the funding and support [7].

To help countries develop policies to enhance efficiency, it is important to quantify efficiency of the national health system, and examine drivers of the efficiency. Implementation of such studies dates back to early 2000, when Murray and Frenk proposed a framework on measurement health system performance[8]. World Health Report 2000 later ranked countries’ performance based on five dimensions encapsulating health outcomes, responsiveness and health financing [9], and generated much interest and debate on evaluating efficiency of national health systems[10]. Since then, several global or regional studies that examine efficiency of providing health or education service using either parametric (e.g. stochastic frontier analysis [SFA]) or non-parametric approaches (e.g. data envelopment analysis [DEA]) have been conducted [11–17]. Studies generally found wide variation in both the efficiency of national health systems in achieving health goals, and the impact of social economic status on efficiency (e.g., inequality of income, and gross domestic product [GDP] per capita)[13].

However, prior studies have rarely investigated the impact of health financing and governance on countries’ health system performance. As countries endeavor to move towards UHC, many have implemented or are undertaking health care reforms on health financing and governance. For example, in 2012, USAID initiated a global health finance and governance project, focusing on health financing and governance issues of the health care system in LMICs and working with recipient countries to mobilize new resources for health and identify strategies to improve the efficiency of care delivery [18].

Given the scarcity of studies on linkage between health financing and governance and health system efficiency, this study is built on the methodology applied in prior efficiency studies of national health systems, and uses more recent data to update the analysis. More importantly, this study includes health system-related factors, such as health financing, in the analysis to quantify their impact on efficiency. In this study, we use DEA to estimate the efficiency of health care systems in 173 countries from 2004 to 2011, examining the variation and change of performance over the eight years. In addition, we construct regression models to examine drivers of health system’s performance, aim to provide insights on health system factors in improving performance of health systems.

## Materials and methods

This study followed a classical framework of economic analysis of efficiency using a two-step process: in the first step efficiency of national health systems were evaluated with direct inputs and outputs, and in the second step econometric models were applied to explain the efficiency [15]. Thus this study considered three sets of variables from each country: (1) direct inputs of health system, (2) direct outputs of the programs, and (3) contextual factors affecting the efficiency.

Similar to other studies, we used health spending per capita as input for the DEA model. The data were obtained from Word Bank Development Indicators as the input for the DEA model [19]. To allow comparison of health expenditure across countries, we standardized health expenditures into 2012 international dollars (I$) after adjusting for purchasing power parity (PPP) and inflation. Literature also shows that education is another important input of health production [11]. We attempted to use secondary school enrollment rate as a measure of education for the model, but found the data on this indicator too limited. Therefore health spending per capita was the only input for the analysis.

As to outputs, we selected infant mortality rate (IMR), under 5 mortality rate (U5MR), and life expectancy (LE) as the key output measurements of a health system. We would like to have included maternal mortality rate (MMR) as an output measurement, but the data on MMR were not publicly available for years 2004 to 2011, and thus MMR was excluded from the model. Under DEA, it is assumed that the larger the value of outputs, the higher the production of decision making units (DMUs). IMR and U5MR are negative indicators and violate the assumption. Hence, we transformed IMR and U5MR into positive indicators, in the same direction of health expenditure. To compute the DEA, we used ln1000 –ln(IMR) or ln1000—ln(U5MR) as outputs for those two indicators on mortality in the efficiency score calculation.

Regarding potential contextual factors explaining efficiency of national health systems, four aspects have been highlighted by the World Bank to improve the efficiency of funding: 1) macroeconomic status, 2) social and cultural factors, 3) infrastructure and human resources, and 4) institutional and policy environment [20]. From the available data, we included indicators on economic and demographic characteristics, health financing mechanisms, and governance as key potential determinants of technical efficiency in the regression analysis.

In this study, the potential economic and demographic variables were: (1) gross national income (GNI) per capita in PPP; (2) HIV/AIDS prevalence for ages 15 to 49; and (3) percentage of urban population as a measurement of urbanization.

The potential health financing variables were: (1) private prepaid plans as a percentage of total health expenditure (THE); (2) out of pocket payments as a percentage of THE; (3) social security expenditure as percentage of general government expenditure; (4) general government expenditure on health as a percentage of THE; (5) external health expenditure as share of THE; (6) commitment to health, measured by the share of general government health spending out of total central government expenditure; and (7) THE as a percentage of GDP.

The potential variables on governance were: (1) voice and accountability, which measures “the degree to which citizens participating in government affairs, and freedom of expression, freedom of association and a free media”; (2) political stability and absence of violence, which measures “perceptions of the likelihood of political instability and/or politically motivated violence, including terrorism”; (3) government effectiveness, which measures “the quality of public services, the quality of the civil service…the quality of policy formulation and implementation”; (4) regulatory quality, which measures “the ability of the government to formulate and implement sound policies and regulations that permit and promote private sector development”; (5) rule of law, which measures “the extent to which agents have confidence in and abide by the rules of society”; and (6) control of corruption, which measures “the extent to which public power is exercised for private gain”[21]. Each indicator on governance had a score ranging from -2.5 to +2.5, with a higher score indicating better performance. These indicators were used widely in econometric models [22–24].

Data on health expenditure and the three outputs (IMR, U5MR, and LE) were obtained from World Bank dataset [19]. Any missing values were imputed. If a country had complete data, eight data points would be presented for each variable (2004–2011). Because of uncertainty on the reliability of data at the country level, we imputed data only for countries with at least five data points for each variable. We interpolated or extrapolated missing values using a log linear function, using the following formula: ln(value) = a + b (year). No imputation was conducted for IMR U5MR and LE, as data were complete. For health expenditure, 0.5% data points were imputed.

Data on potential determinants were obtained from databases constructed by the World Bank and the World Health Organization [1, 19]. If data were missing, the same imputation approaches as that for the input and outputs were applied. Data on governance were complete, and no imputation was conducted. For indicators on health financing, 0.88% data points were imputed. For variables related to economic and demographic variables, 2.46% data points were imputed for GNI/capita, 0.18% for urbanization, and 0.22% for HIV prevalence. The final dataset covered 173 countries from 2004 through 2011.

In DEA models used to evaluate the efficiency of health systems, performance was defined as the ratio of weighted outputs to weighted inputs. We generated two sets of efficiency scores for the countries in the study. The first set of efficiency scores were produced by comparing each country with the best country(ies) in the same year (Separate DEA model), and thus eight DEA models were implemented for the eight years of data. We also pooled the data for all countries over the eight years, and generated another set of efficiency scores (Pooled DEA model). In this model, each country was compared to the best performers among the eight years using one production frontier. All DEA models were implemented using input-orientation and variable return to scale developed by Charnes et al.[25]. Unlike conventional parametric approaches, DEA is capable of modeling production with multiple inputs and multiple outputs by comparing each decision making unit to a production frontier defined by the best performers from data, and has been applied in many settings to evaluate the performance of various types of organizations and programs[26].

To examine the impact of contextual variables on efficiency of health care systems, we constructed Tobit models as the second stage analysis [24, 27, 28], using the pooled efficiency scores as the dependent variable and contextual factors as independent variables. Because the indicators on health financing and governance were highly correlated, factors analysis was carried out to select the most representative indicator within each factor to be included in the regression models. We found that two factors from financing variables could explain more than 85% of the information on health financing, and we selected one indicator from each factor (two indicators in total for health financing) for the regression analysis. Similarly, we found that one factor from governance indicators could explain more than 90% of the information, and thus we included only one indicator on governance in the regression to explain the efficiency. The selection of the variable in the regression from each factor was based on the magnitude of factor loadings. For health financing indicators, social security expenditure as a percentage of THE and government health spending as a percentage of the total government budget were selected for the regression analysis, while only rule of law was selected among the governance variables.

We constructed five models by adding independent variables subsequently. The full model is expressed as

$$\ln\left({efficiency}_{it}\right)=\beta_{0}+\beta_{1}{lnGNIPC}_{it}+\beta_{2}{lnGNIPCsq}_{it}+\beta_{3}{Urbanization}_{it}+\beta_{4}{HIVprevalence}_{it}+\beta_{5}{SSEtoTHE}_{it}+\beta_{6}{GHEtoTGB}_{it}+\beta_{7}{RoL}_{it}+\alpha_{i}+\epsilon_{it}$$

Where i represents ith countries, and t represents tth year; GNIPC denotes gross national income per capita, GNIPCsq the square of GNIPC, urbanization the percentage of population living in urban area, HIVprevalance the prevalence of HIV in the country, SSEtoTHE the ratio of social security expenditure to total health expenditure, GHEtoTGB the ratio of government health expenditure to total government budget, RoL the rule of law, α_i_ the individual impact of ith country, and ε_it_ the random noise.

The DEA was conducted with DEA-solver 5.0 (Saitech Inc. New Jersey) and the regression model was implemented with STATA 12 (StataCorp LP, Texas).

## Results

Table 1 shows the descriptive results of the inputs and outputs in this study. Over the eight year period, the health spending per capita increased by 20.13% (with annual growth rate of 2.33%), rising from I$925.33 in 2004 to I$1112.62 in 2011. As a consequence, IMR was reduced by 21.24% (with annual reduction rate of 2.94%), from 34.59 in 2004 to 27.24 per 1,000 live births in 2011; U5MR was reduced by 24.45%, from 49.52 in 2004 to 37.41 per 1,000 live births in 2011; and life expectancy increase by 2.25 years, from 67.73 in 2004 to 69.98 years old in 2011, with annual growth rate of 0.41%.

**Table 1:** Inputs and outputs of health system.

Year	Heath Spending/Capita (PPP)	Infant Mortality Rate	Under 5 Mortality Rate	Life Expectancy
2004	925.33±1289.25	34.59±30.97	49.52±50.85	67.73±10.1
2005	940.27±1295.89	33.43±30.16	47.60±49.15	68.01±9.99
2006	974.82±1339.87	32.30±29.36	45.76±47.47	68.37±9.92
2007	1010.72±1368.30	31.21±28.57	43.95±45.80	68.69±9.78
2008	1050.22±1411.15	30.18±27.84	42.24±44.19	69.00±9.61
2009	1113.06±1462.57	29.14±27.05	40.56±42.59	69.33±9.50
2010	1109.44±1472.26	28.25±26.43	39.47±42.17	69.65±9.40
2011	1112.62±1473.33	27.24±25.56	37.41±39.49	69.98±9.35
Annual growth rate	2.33%	-2.94%	-3.44%	0.41%

N = 173. Notation: PPP denotes purchasing power parity.

**Fig 1:**
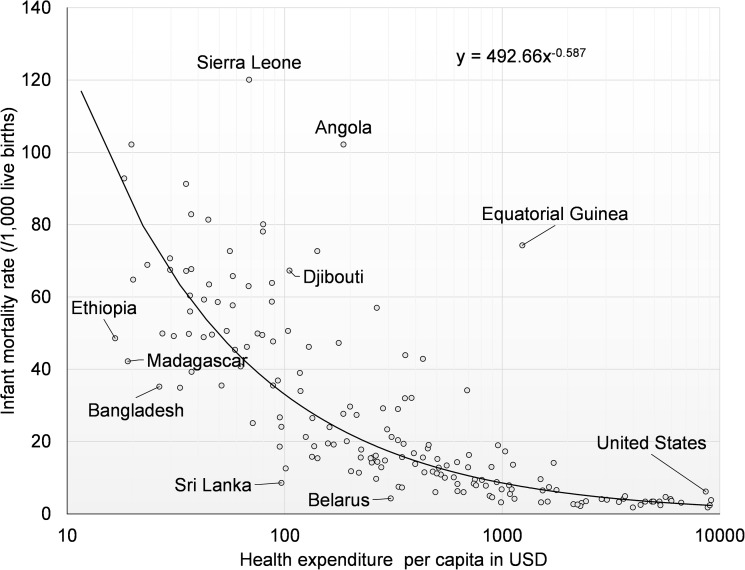
Health expenditure per capita and infant mortality rate in 2011.

There is a clear relationship between health expenditure and IMR, as shown in Fig 1 for 2011. On average, health spending was negatively associated with IMR. With the increase of health spending, IMR dropped substantially and then it leveled off. In the countries where health spending was less than $100, it is likely that an increase of health spending would yield substantial health benefits. The health spending elasticity was estimated at -0.59, indicating that if health spending per capita increases by 1%, IMR would drop by 0.59%.

Fig 1 also shows the wide variation of performance of countries in achieving this indicator: IMR varied substantially for a given amount of health expenditure per capita. For example, among countries with health expenditure around $100, IMR ranged from 8.6 in Sri Lanka to 67.3 per 1,000 live birth in Djibouti. Other countries with good performance for IMR were Ethiopia, Madagascar, Bangladesh, and Belarus.

**Fig 2:**
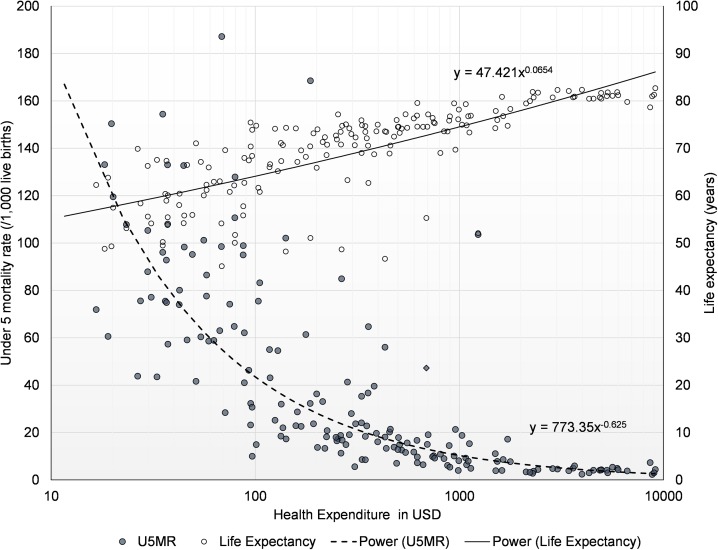
Health expenditure per capita and under 5 mortality rate and life expectancy in 2011.

Fig 2 shows the relationship between health expenditure per capita and U5MR and life expectancy. Clearly, there was a negative relationship between health expenditure per capita and U5MR, with an elasticity of -0.62. It suggested that 1% increase in health expenditure per capita was associated with 0.62% reduction in U5MR. Health expenditure per capital was positively associated with life expectancy, with an elasticity of 0.065. Similar to Fig 1, Fig 2 also demonstrated wide variation of performance of health system in achieving low U5MR and long life expectancy. The wide spread of data points in terms of health outcomes were mostly distributed where health spending per capital was below $316/capita (10^2.5), which suggested that LMICs, in general, had more room to improve the efficiency.

A separate DEA model used the eight periods of data to generate eight sets of efficiency scores. However, none of the eight sets of efficiency scores could be compared as they were based on different production frontiers. Our results showed that during 2004–2011, the mean efficiency score was 78.9% (Table 2). This suggests that the countries could save 21.1% of health expenditure per capita to achieve the same level of health outcomes if countries performed as well as their peers. The countries with efficiency score of 100% were Bangladesh (2004–2011), Iceland (2004–2011), Israel (2006–2007), Japan (2004–2011), Sri Lanka (2004–2011), Madagascar (2008–2011), Singapore (2004–2010), Syria (2006–2011) and Vietnam (2004–2011).

**Table 2:** Efficiency of health system in 2004–2011.

**Year**	**Separate DEA efficiency**	**Pooled DEA efficiency**
2004	0.723±0.194	0.707±0.185
2005	0.759±0.154	0.712±0.183
2006	0.778±0.13	0.719±0.181
2007	0.791±0.115	0.724±0.177
2008	0.805±0.108	0.729±0.173
2009	0.804±0.109	0.733±0.171
2010	0.819±0.105	0.743±0.169
2011	0.832±0.103	0.752±0.169
Annual growth	-	0.77%

Notation: DEA denotes data envelopment analysis.

When we pooled the eight years of data together using one production frontier, we found that there was improvement in the efficiency of national health systems over the eight-year period. The efficiency improved from 70.7% in 2004 to 75.2% in 2011, with an annual growth rate of 0.77% (Table 2).

**Fig 3:**
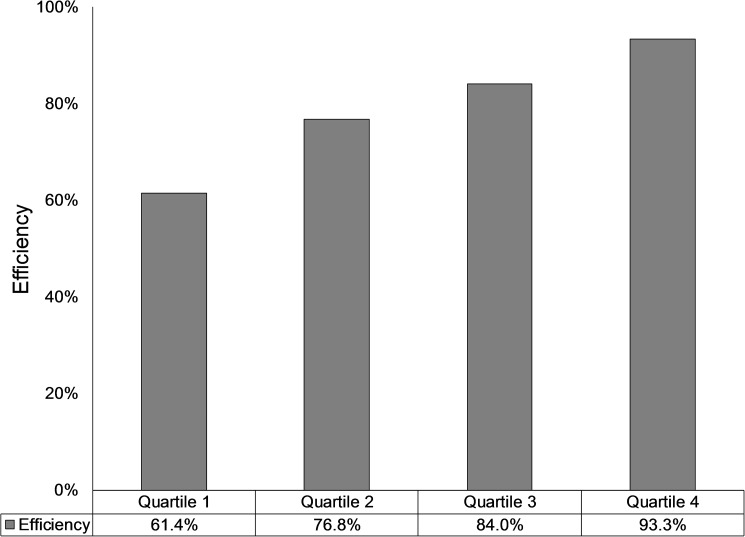
Efficiency of national health systems by quartiles (2004–2011).

Similar to the results from separate health outcomes, when combining the three health outcomes together using DEA, we found that efficiency varied substantially among countries. Fig 3 illustrates the average efficiency of health systems by quartiles. The mean efficiency for quartile 1 (the worst fourth of observations) was 61.4%, 76.8% for quartile 2, 84.0% for quartile 3, and 93.3% for quartile 4 (the best fourth of observations). For countries in the lowest quantile, much improvement could be done to enhance the efficiency, with a potential of saving 38.6% of health expenditure per capita. In 2011, Equatorial Guinea, Botswana, South Africa, Swaziland, Gabon, Trinidad and Tobago, Angola, The Bahamas, Micronesia, and Lesotho were in the list of low performance countries.

Table 3 shows the regional differences of the efficiency of national health systems. We found that Africa had the lowest efficiency, with an average efficiency of 67%. The region with the highest efficiency is Western Pacific, which had an average efficiency of 86%.

**Table 3:** Efficiency of health systems in 2011 by WHO regions.

**Region**	N*	**Mean ± SD**
Africa	43	0.67±0.17
Americas	31	0.78±0.07
Eastern Mediterranean	20	0.79±0.09
Europe	49	0.85±0.08
Southeast Asia	9	0.87±0.10
Western Pacific	21	0.86±0.09

*N denotes the number of countries in the analysis.

Table 4 shows the five models used to examine the impact of social economic status, urbanization, HIV/AIDS prevalence, and health spending, as well as governance on efficiency. All variables used in five models were statistically significant (p<0.05), and hence we focused on the results from model 5. The results from model 5 show that social economic status affected the efficiency. As the economic status improved, the efficiency of the health system improved until GNI/capita reached the level of I$10,097; then the efficiency declined as the economic status grew. Urbanization was statistically significant and positively contributed to the efficiency of national health systems. A 1% point increase in urbanization was associated with 0.4% increase in the efficiency of national health system. HIV/AIDS burden reduced the efficiency of the health system. If HIV prevalence increased by 1 percentage point, the efficiency would be reduced by 5.9%. Health financing mechanisms played a significant role in determining the efficiency of health care systems. A higher share of social health insurance spending was associated with higher performance of the health system: a 1% point increase of the share of social security expenditure was related to 1.9% increase of the health system efficiency. Surprisingly, the share of government health spending of the total government budget was negatively associated with the health system efficiency. Stronger rule of law was associated with higher performance of the health system in the country.

**Table 4:** Determinants of the performance of national health systems.

Independent Variables	Model 1	Model 2	Model 3	Model 4	Model 5
Log of GNI per capita (PPP)	0.790***	0.733***	0.906***	0.939***	0.922***
Square of log of GNI per capita	-0.038***	-0.037***	-0.048***	-0.051***	-0.050***
Urbanization (%)		0.005***	0.003*	0.004*	0.004**
HIV prevalence (%)			-0.059***	-0.059***	-0.059***
Share of social security expenditure among total health spending (%)				0.019***	0.019***
Share of government health spending among total government budget (%)				-0.017***	-0.017***
Rule of law					0.044**
Constant	-4.300***	-4.179***	-4.567***	-4.676***	-4.585***

Notation: GNI denotes gross national income; PPP denotes purchasing power parity. Statistical significance is indicated by

* (p<0.05)

** (p<0.01) and

***(p<0.001)

## Discussion

We found that the average efficiency of the national health systems from separate DEA was 78.9%, indicating a potential saving of 21.1% of total health spending per capita to achieve current health status for children and the entire population if all the countries performed as well as their peers. Given that input-oriented efficiency is highly correlated with output-oriented efficiency, the result also suggests that the weighted health outcomes would be increased by 26% if the funding were appropriately allocated and used.

The efficiency gap between the top 25% countries and the bottom 25% countries is substantial (93.3% vs. 61.4%). The result not only shows wide variation of health resources available across countries around the world, but also reflects the substantial gaps in terms of health outcomes. In LMICs, particularly Africa, the shortage of financial resources for health and high disease burden, in conjunction with low efficiency, suggests that those countries have limited capacity to transfer available funding to health benefits for the targeted population, which further slows the progress of those countries to move towards internationally committed goals, such as MDGs and SDGs.

Consistent with Grigoli and Kapsoli’s study[17], we also found that overall inefficiency was highest in Africa, while western and Asian countries were relatively more efficient. It is worth noting that in the countries with similar economic status, the efficiency of the health system can be vastly different. Taking Croatia and Equatorial Guinea as an example, where the health spending per capita in 2011 was $1137 and $1236 respectively, we found enormous differences in all three health outcomes (life expectancy: 76.8 vs. 52.1; IMR: 4.2 per 1000 live births vs. 74.3; under 5 mortality rate: 4.9 per 1000 live births vs. 103.6). Similarly, we found large differences between South Africa and Lebanon in terms of health outcomes, while both countries had comparable health spending per capita. Thus for countries with low efficiency and low health spending, strengthening the efficiency of health care system is as important as adding more resources through various mechanisms (e.g. strengthening tax collection and obtaining more donor support) to address the health needs of populations. It is likely that large gains in health outcomes can be realized by strengthening the efficiency of health spending. As many LMICs are undergoing health care reform, developing strategies to enhance health system efficiency should be emphasized in the health care reform agenda [6].

Although many LMICs face challenges in achieving high performance in using health fund, fortunately, we found that health system performance has improved over time among many of them. This improvement of the efficiency over time provides the governments more opportunities to allocate the funding that could be saved on enhanced efficiency to other important health areas (i.e. AIDS), and expand the fiscal space for disease control [29]. Such a reallocation of funding and reprioritization of health interventions may further improve the efficiency of the health system and yield better health outcomes.

Health financing mechanisms are highly associated with health system efficiency [30]. In this study, we found a statistically significant impact of social security spending on the health system efficiency. A 1% point increase of the share of social security spending was related to a 1.9% increase of the health system efficiency. The finding echoes what WHO has been advocating on social health insurance in developing countries: to provide risk and financial protection to poor populations in order to achieve better health outcomes. Since 2010, WHO and governments worldwide have endeavored to find the best approach to meeting populations’ health needs, and work hard to achieve universal health coverage (UHC). It is clear that UHC cannot be attained without a well-functioning and efficient health system [31]. What is interesting from this study is that when more and more populations are included in the social security, the health system becomes more efficient not only in delivering health services, but also in achieving desired health outcomes, resulting in faster progress towards UHC. The efficiency gains from social health insurance may be due to several reasons: (1) In order to fulfill the criteria of good governance, the administration of health insurance funds often separates from Ministries of Health, which creates spaces for using an “indirect” service provision model, a model that relies more on market competition for efficiency than central planning and bureaucratic rules [30]; (2) in countries where social health insurance is in place, strategic payment mechanisms are likely established, such as capitation for outpatient care or diagnosis related groups for inpatient care, rather than fee for services, which enhance the efficiency of using available funds; and (3) with a basket of funding from social health insurance, purchasing agency(ies) could exert its(their) market power to purchase services from providers with lower payment rates.

Interestingly, government spending on health as share of the government budget is negatively associated with the performance of the health system. This indicator may convey mixed indications on efficiency. On one hand, this indicator signals the commitment of government in improving health and is positively associated with the performance [24]. On the other hand, after controlling for social security spending as share of total health spending, this indicator may indicate the degree to which the service delivery system is organized using a central planned model, which often has a low efficiency. Although it is possible that governments could also use a health budget strategic contract with public and private providers, directly financing public providers is the most common approach in low- and middle-income countries when using a government budget to fund health. Thus, this indicator should be interpreted with caution. Further investigation of how service delivery systems are organized and financed in countries would be helpful to understand the relationship between government spending and efficiency by cases.

As expected, strong governance is another factor contributing to high performance of a country’s health system. Because of a high correlation among governance indicators (voice and accountability, government effectiveness, rule of law, control of corruption, political stability, and regulatory quality), we considered the indicator of rule of law to represent a collection of governance characteristics of a country. Governance affects the efficiency in multiple ways. Firstly, studies showed that governance could affect the inputs of health services, such as public spending and quality of personnel. Rajkumar investigated the role of governance in the relationship of public spending and U5MR, and found that in countries which are very corrupt or have a very ineffective bureaucracy, public health spending will be ineffective at the margin [32]. Secondly, good governance provides an enabling environment for health services delivery. Governance shows the capacity of government and non-government to achieve national health goals in a country, which is manifested by good implementation of institutions and rules, sound structure of administrative bodies, and strong coordination capacity. All these elements are instrumental in ensuring effective health care delivery. If governance issues are not addressed, returns to investments in health are low [33, 34].

This study has a few limitations to be acknowledged. First, there is missing information of the data, necessitating our imputing missing values. For key variables, data are complete for IMR, U5MR and LE; health expenditure was missing 0.5% of values (7 out of 1,392), which is a relatively small share. Secondly, in constructing the DEA model, we had wanted to include education as an additional input and MMR as an additional output for the health system. However, due to constraints of data, these two variables are excluded from our analysis. Thus the results from the DEA models are not fully able to adjust the quality of human resource in producing health outcomes, although education is correlated with governance (the correlation coefficient is 0.66 based on available data), which was used as a regressor in the second stage of DEA. Thirdly, since governance indicators are highly correlated, we had to select the one most representative indicator to include in the regression model—rule of law in this study. The interpretation of this variable should go beyond “rule of law” as it represents more the overall picture of governance in a country. In spite of these limitations, given the small number of missing values in the data set and high correlation between education and governance, some concerns over the limitations are mitigated.
